# Psychological Processes of Telecommunications Fraud: Development of a Three-Stage Framework and Cross-Sectional Quantitative Elaboration in a Mixed Methods Study

**DOI:** 10.2196/87395

**Published:** 2026-08-13

**Authors:** Yujie Huang, Jun Gao, Yuhong Zhou, Qinghua He, Xuemei Gao

**Affiliations:** 1 Psychological Research and Consultation Center, Southwest Jiaotong University, No. 999, Xi'an Road, Pidu District, Chengdu, Sichuan, 611756, China, 86 17383011768; 2 Faculty of Psychology, Southwest University, Chongqing, China; 3 Nan'an District Branch of Chongqing Municipal Public Security Bureau, Nan'an District, Chongqing, China

**Keywords:** telecommunication fraud, gullibility, emotion regulation, critical thinking, self-control, mixed methods

## Abstract

**Background:**

Telecommunications fraud causes financial and psychological harm, yet how individuals who experienced fraud retrospectively describe progression from initial trust to emotional escalation and reduced behavioral control remains unclear.

**Objective:**

This study aimed to develop a stage-based psychological framework for telecommunications fraud and examine whether person-level cross-sectional associations provide quantitative elaboration.

**Methods:**

Study 1 used grounded-theory analysis of semistructured interviews with 71 individuals who experienced telecommunications fraud; 46 interviews informed model development and 25 assessed theoretical saturation. Study 2 analyzed 4722 university-based survey responses from Chongqing, including 409 students with confirmed fraud experience. A balanced case-control sample comprised all 409 participants with confirmed fraud experience and 409 randomly selected participants without reported fraud experience. Two-tailed independent-samples *t* tests, adjusted logistic regression, and 10 nonoverlapping resampling iterations examined correlates of victimization. Among defrauded people, 2 theory-specified PROCESS Model 6 analyses with 5000 bootstrap resamples estimated cross-sectional indirect associations involving Truth-Seeking or Cognitive Maturity, Gullibility, Difficulties in Emotion Regulation, and Brief Self-Control Scale (BSCS) scores.

**Results:**

Study 1 yielded a 3-stage interpretive framework of retrospective narratives: Credulity Priming, Affective Manipulation, and Behavioral Dyscontrol. Desire and fear loops, as well as overexpectation events, were characterized by a narrated escalation. In study 2, defrauded people had lower Truth-Seeking and Cognitive Maturity and higher Gullibility, Difficulties in Emotion Regulation, and BSCS scores (indicating poorer self-control) than sampled nondefrauded people. In adjusted analyses, higher Gullibility was associated with higher odds of victimization, whereas higher Truth-Seeking was associated with lower odds; the interpretation of these adjusted correlates was based primarily on their consistent reproduction in the student-only sensitivity models. Among defrauded people, both PROCESS models yielded statistically significant model-specified cross-sectional indirect association estimates, but concurrent postevent measurement precluded temporal or causal inference. Across studies, convergence was the strongest for trust-related appraisal; evidence for affective and behavioral domains was complementary rather than confirmatory.

**Conclusions:**

Retrospective narratives supported a 3-stage interpretive framework, while cross-sectional quantitative findings most consistently linked lower Truth-Seeking and higher Gullibility to victimization status in this sample. These findings motivate prospective evaluation of stage-sensitive prevention strategies; the proposed temporal sequence and intervention implications require direct testing in more diverse populations.

## Introduction

Fraud is a long-standing social problem [1,2], but digital communication has expanded its speed, scale, and remote reach [3]. Telecommunications fraud uses telephones, messaging, email, and social platforms to induce financial transfers or the disclosure of sensitive information [4], making it a digital public health concern [5-8]. Beyond financial loss [9,10], victimization can produce shame, self-blame, fear, posttraumatic stress, and social withdrawal [11-13]. Psychosocial vulnerabilities, such as loneliness, may shape exposure [14], but a trauma-informed framing recognizes the coercive conditions of victimization rather than reducing it to personal carelessness.

Fraud compliance is better understood as an interactional process than as a single error in judgment. Victim-survivor research documents pervasive distress and unmet support needs [15], while analyses of Chinese cellphone fraud show how repetition, interruption, and threatening language induce panic [16]. Together with cumulative demands and time pressure, these tactics can narrow attention and increase cognitive load, reducing opportunities to pause or verify and promoting gradual compliance [2,16]. Under high arousal, affect can constrain deliberation and impair appraisal [17]. After money, time, or trust has been invested, promises of recovery can turn prior commitment into sunk-cost escalation [2]. The digital environment is therefore part of the risk architecture, enabling persistent pressure across communication and payment channels.

Dual-process theory offers a useful account of why these pressures matter. System 1 is fast, intuitive, and affect-driven, whereas system 2 is slower, deliberate, and cognitively demanding [18]. Under urgency and arousal, heuristic processing may dominate judgment [19]. Person-level characteristics such as critical thinking, gullibility, trust, and emotion regulation reflect dispositional susceptibility [20-22], whereas scammer-controlled authority cues and other psychologically structured tactics characterize the immediate interaction [23,24]. This distinction between dispositional susceptibility and situational coercion shifts the explanation away from attributing fraud to individual deficiencies and toward the interaction between person-level susceptibility and situational coercion across a fraud episode.

Prior research suggests that fraud may unfold sequentially [25], yet dual-process theory does not fully represent trust formation, identity manipulation, emotional reversals, or changes across an episode. A stage-based interpretive framework could organize these dynamics and generate testable hypotheses about when digital safeguards might be most useful [8,26].

Cybersecurity research has operationalized broad preventive capacities, including awareness, privacy vigilance, trust calibration, and resilience [27-30]. These measures capture general readiness rather than psychological dynamics once a fraudulent interaction begins. The present event-level framework therefore complements rather than replaces awareness- and resilience-based approaches.

In study 1, we conducted semistructured interviews with individuals who experienced fraud identified via a contact list provided by the Chongqing Public Security Bureau. Grounded-theory analysis of these retrospective accounts identified recurring patterns of trust formation, affective manipulation, and behavioral dyscontrol, which were integrated into a 3-stage event-level interpretive framework.

Study 2 linked person-level dispositions to conceptually corresponding event-level processes retrospectively identified in study 1. Truth-Seeking and Cognitive Maturity corresponded to reflective appraisal, Gullibility to trust calibration, Difficulties in Emotion Regulation to affective vulnerability, and lower Brief Self-Control Scale (BSCS) scores to better self-control. Because the quantitative data were cross-sectional, these measures did not capture within-episode states; the theory-specified indirect associations summarized model-based covariance rather than temporal or causal ordering. They therefore provided only cross-sectional quantitative elaboration of the qualitative framework.

## Methods

### Ethical Considerations

The study was conducted in accordance with the Declaration of Helsinki. Ethical approval for this mixed methods research (encompassing both study 1 and study 2) was granted by the Institutional Review Board of Southwest University (approval number: H25090).

Informed consent was obtained from all participants prior to their involvement. For study 1, the research team obtained explicit informed consent before each semistructured interview. For study 2, electronic informed consent was obtained from all survey respondents on the Wenjuanxing platform before they could access the questionnaire.

Strict privacy and confidentiality measures were enforced throughout the research. In study 1, to protect privacy and data security, all interview transcripts were cleaned and completely deidentified prior to analysis. All raw data and contact lists were accessible exclusively to the core research team, and all team members signed formal confidentiality agreements to ensure no data were disclosed. In study 2, survey data were collected anonymously without recording any personally identifiable information.

Regarding participant compensation, interviewees in study 1 received 30 RMB (US $1=6.7719 RMB as of July 24, 2026) for their time, and survey participants in study 2 received a small monetary reward upon successfully passing the attention checks. The qualitative component was reported in accordance with the COREQ (Consolidated Criteria for Reporting Qualitative Research; Checklist 1). The web-based survey was reported in accordance with the CHERRIES (Checklist for Reporting Results of Internet E-Surveys; Checklist 2).

### Study 1: Qualitative Phase

#### Participants

The textual data analyzed in this study were derived from semistructured interviews with individuals who experienced telecommunications fraud. The Chongqing Public Security Bureau assisted with initial participant recruitment, allowing potentially eligible individuals to verify that the research invitation was legitimate rather than another fraudulent contact. The bureau did not participate in informed consent, interviewing, or data analysis. The cases covered incidents occurring between January 1 and March 31, 2024.

To ensure data quality and relevance, respondents were selected based on strict inclusion criteria: (1) adults aged 18 or older, (2) officially registered as having experienced telecommunications fraud within the specified timeframe, and (3) capable of and willing to clearly articulate their victimization process. The research team provided the bureau with a detailed, standardized recruitment script explaining the study’s academic purpose, its independence from official investigations and case-handling procedures, and the safeguards for participant privacy. The script stated that participation was entirely voluntary; individuals could decline or withdraw at any time without giving a reason or affecting any legal, administrative, or fraud-related support procedure; and individual interview responses would not be disclosed to public security personnel. The research team subsequently obtained informed consent and conducted the interviews. After screening out those who declined to participate or provided incomplete accounts, 71 interviews with individuals who experienced fraud were retained. Prior to the interviews, no relationship had been established with the participants. The interviews were conducted individually via telephone to ensure participants’ privacy and comfort. Each interview lasted approximately 30 minutes. With participants’ explicit consent, the interviews were audio-recorded and subsequently transcribed verbatim for analysis.

Interviews followed a semistructured, event-timeline guide (Appendix A10 in Multimedia Appendix 1). Participants were first invited to recount the episode in their own words; interviewers then used optional probes covering initial contact and perceived credibility, escalation and verification, cognitive and emotional changes, turning points, behavioral compliance and difficulty stopping, termination, and postevent effects.

#### Data Analysis

##### Overview

To ensure methodological rigor and transparency, NVivo 20.0 (QSR International Pty Ltd) was used to enhance the efficiency and traceability of text coding. After cleaning and deidentification, all materials were uniformly imported into NVivo and analyzed following the classic grounded-theory procedure in 3 stages. Coding was conducted by the first author and reviewed by a second researcher with qualitative research experience. An initial subset of transcripts was independently coded by both researchers during the open-coding phase, after which discrepancies in code labels, category boundaries, and stage assignments were discussed until consensus was reached. A running codebook was refined iteratively to document code definitions, inclusion and exclusion criteria, representative excerpts, and decisions about category boundaries and stage assignments. The remaining transcripts were coded using the revised codebook, and ambiguous cases were revisited through team discussion to maintain conceptual consistency.

##### Open Coding

Based on an initial close reading of the interviews, researchers analyzed the texts sentence by sentence and paragraph by paragraph, identifying and labeling key phrases and semantic units related to the fraud process, emotional changes, and behavioral responses.

##### Axial Coding

Axial coding is the process of relating subcategories to their primary categories by systematically analyzing the conditions, actions, and consequences associated with a phenomenon [31]. The initial codes were then organized into higher-order categories by examining their properties and dimensions, and logically reassembled according to the condition-action-consequence paradigm.

##### Selective (Core) Coding

Building on the axial coding, a final set of core categories was developed to integrate the analysis.

**Figure 1.**
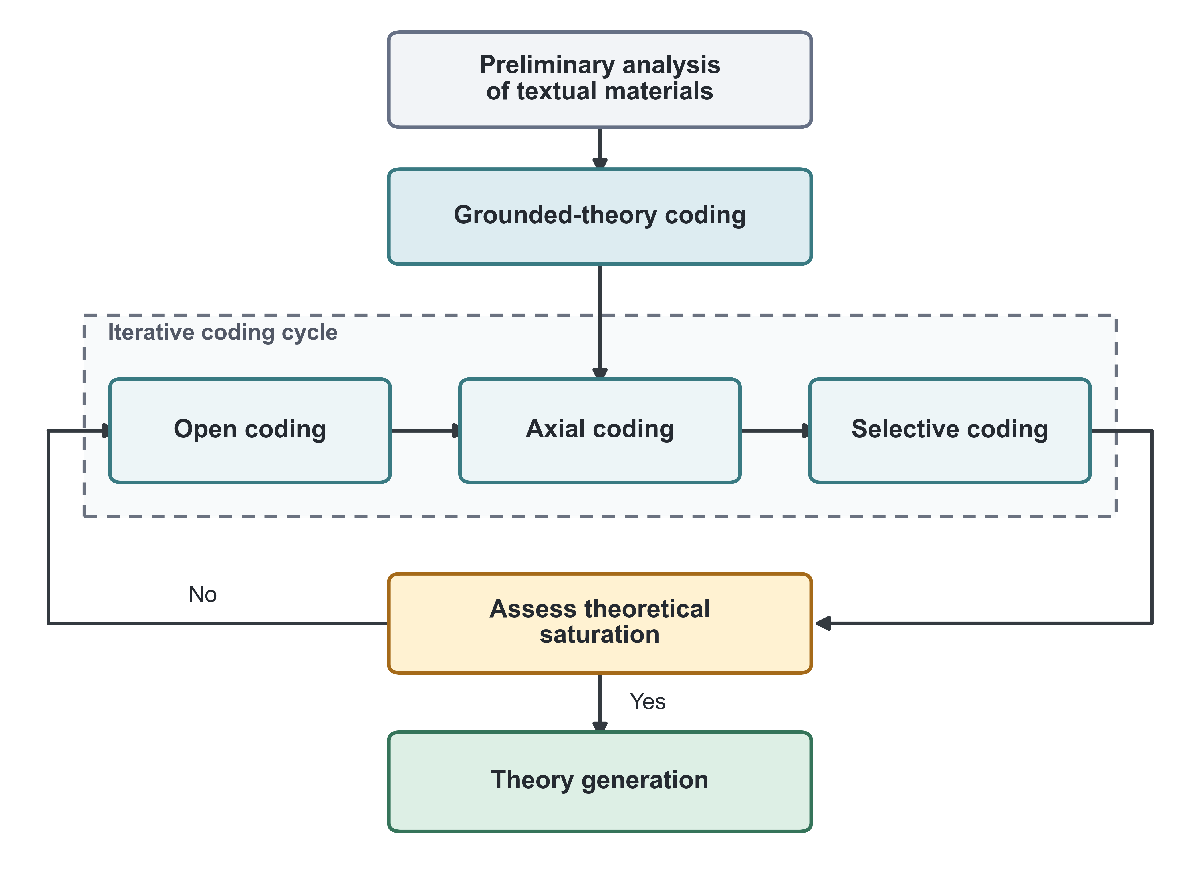
Grounded-theory workflow for model development and theoretical saturation testing in study 1.

The running codebook and detailed outputs from the open, axial, and selective coding stages provide an audit trail from initial codes to the final categories (Appendices A1-A3 in Multimedia Appendix 1), allowing the main text to focus on the integrated theoretical model. The qualitative analytic workflow is summarized in Figure 1.

### Study 2: Quantitative Phase

#### Participants

Participants were recruited by cluster sampling at a university in Chongqing, and the data were collected through the Wenjuanxing online platform between April and June 2025. The survey was advertised through internal university WeChat groups, and the platform limited access to one submission per IP address and WeChat account. Of 5323 responses, 4921 remained after excluding incomplete questionnaires and unrealistically short completion times. Among the 608 participants who self-reported telecommunications fraud victimization, 199 failed embedded attention checks and were excluded.

#### Materials

##### Brief Self-Control Scale

We used the BSCS to assess general self-regulatory capacity. The scale contains 13 items rated from 1 (not at all like me) to 5 (very much like me). In all analyses, higher BSCS scores indicate poorer self-control [32]. Cronbach α was 0.816 in the present study.

##### Gullibility Questionnaire

We used the Gullibility Scale developed to measure the tendency to accept false premises in the presence of unreliable information. The questionnaire comprises 12 items rated on a 1 (strongly disagree) to 5 (strongly agree) Likert scale, with higher total scores reflecting higher gullibility [33]. In this study, Cronbach α was 0.741.

##### Critical Thinking Questionnaire

We used the Chinese version of the Critical Thinking Disposition Inventory (CTDI–CV) [34]. The inventory includes 7 dimensions; we selected 2 dimensions most relevant to fraud: Truth-Seeking and Cognitive Maturity. Items are rated on a 6-point Likert scale, where 1 to 6 indicate strongly agree to strongly disagree; higher scores denote stronger dispositions on the respective dimensions. In this study, Cronbach α was 0.824 for Truth-Seeking and 0.908 for Cognitive Maturity, and 0.808 for the total scale.

##### Difficulties in Emotion Regulation Questionnaire

We used the Chinese translation of the Difficulties in Emotion Regulation Scale (DERS) [35]. The scale contains 36 items rated on a 1 (almost never) to 5 (almost always) Likert scale. Participants chose the option that best matched their actual situation; higher scores indicate greater difficulties in emotion regulation. The DERS includes 6 subscales: Nonacceptance of emotional responses, Difficulties engaging in goal-directed behavior, Impulse control difficulties, Lack of emotional awareness, Limited access to emotion-regulation strategies, and Lack of emotional clarity. In the present study, Cronbach α values were 0.882 (Nonacceptance), 0.814 (Goals), 0.835 (Impulse), 0.792 (Awareness), 0.878 (Strategies), and 0.659 (Clarity), with 0.925 for the total scale.

### Data Analysis

The data were analyzed using IBM SPSS Statistics 25.0 and Python 3.9.7. SPSS was used for data cleaning, descriptive statistics, bivariate correlations, and theory-specified PROCESS Model 6 cross-sectional indirect-association analyses. Python was used for the case-control comparison and robustness checks.

To separate descriptive group differences from adjusted correlates of fraud victimization, we first constructed a balanced case-control dataset by randomly selecting 409 nondefrauded people from the larger pool of 4313 nondefrauded people, yielding a 1:1 dataset of 818 participants. Two-tailed independent-samples *t* tests were used to describe unadjusted group differences between defrauded people and nondefrauded people. Binary logistic regression was then conducted to identify variables independently associated with fraud victimization, with age and gender entered as covariates. Fraud victimization was coded as 1=“yes” and 0=“no”; gender was coded as 0=“male” and 1=“female.” Within-defrauded people correlations and theory-specified cross-sectional indirect associations were interpreted separately from these case-control models.

To further test the stability of the case-control findings, we conducted a 10-iteration nonoverlapping resampling robustness check in Python using the pandas, scipy.stats, and statsmodels libraries. Specifically, the nondefrauded people pool was first shuffled with a fixed random seed and then partitioned into 10 mutually exclusive subsets of 409 participants each. In each iteration, the full sample of 409 participants with confirmed fraud experience were compared with one new nonoverlapping subset of 409 participants without reported fraud experience. Two-tailed Welch *t* tests and binary logistic regressions were repeated across all 10 datasets. To further address the comparability concern arising from the fact that all confirmed defrauded people were university students, we conducted additional student-only sensitivity analyses. Specifically, 124 staff respondents were excluded from the pool of participants without reported fraud experience, leaving 4189 eligible students without reported fraud experience. Using this student-only pool, we repeated the 10-iteration nonoverlapping resampling procedure and re-estimated both 2-tailed Welch *t* tests and binary logistic regressions across all 10 matched comparisons. Because all participants with confirmed fraud experience were university students, the student-only models provided the more directly comparable basis for interpreting adjusted correlates.

To examine theory-informed statistical associations within the postevent sample of 409 participants with confirmed fraud experience, theory-specified PROCESS Model 6 analyses were conducted exclusively among the 409 confirmed defrauded people using Hayes PROCESS macro (Model 6). Truth-Seeking and Cognitive Maturity were entered as focal variables in 2 separate models, Gullibility and Difficulties in Emotion Regulation were entered as model-specified intermediate variables, and the BSCS score served as the outcome variable. Because all variables were measured concurrently after victimization, the variable arrangement was specified from the qualitative framework and prior theory; it does not establish temporal order or causality. Model-specified cross-sectional indirect associations were estimated using 5000 bootstrap resamples and 95% CIs.

## Results

### Study 1: Qualitative Findings

#### Sample Characteristics

The final qualitative sample included 71 individuals who experienced telecommunications fraud, comprising 32 men and 39 women, with a mean age of 33.97 (SD 10.25) years. Regarding occupational background, the sample included enterprise employees (n=14), freelancers (n=14), retirees (n=3), and a university student (n=1), as well as individuals from various service and professional sectors, including nurses, teachers, security personnel, and service staff (n=12). The remaining 27 participants did not disclose their occupations. This provided some variation in occupational background within the qualitative sample.

#### Theory Construction

##### Overview

**Figure 2.**
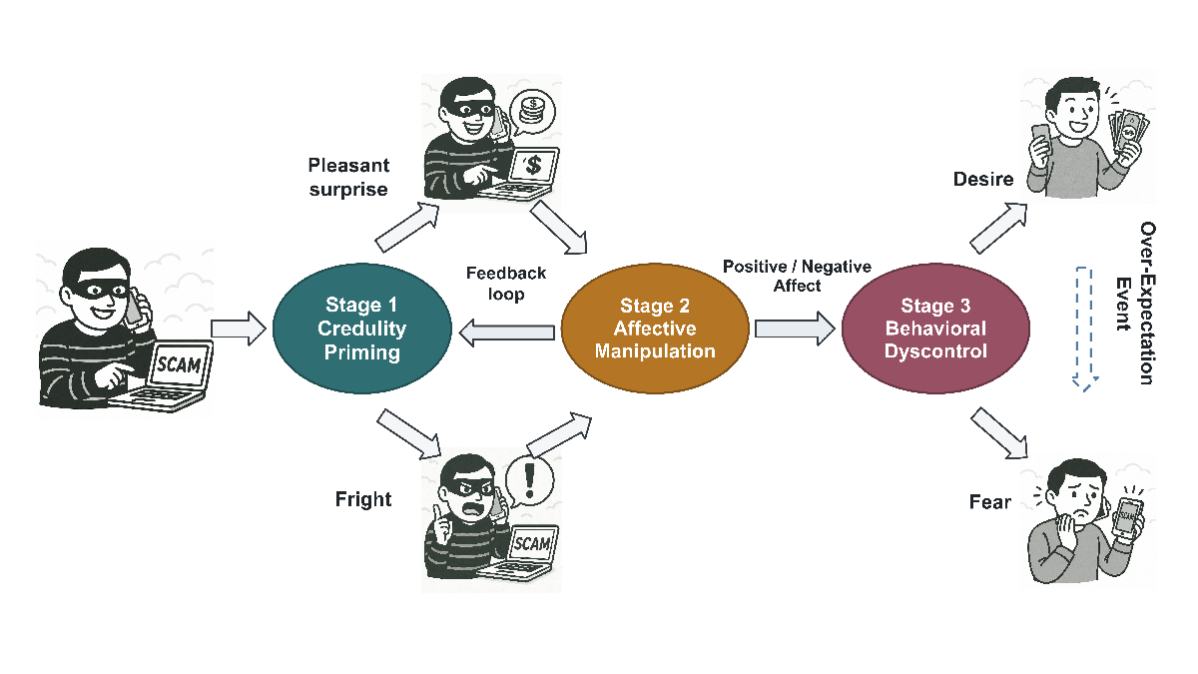
The credulity priming-affective manipulation-behavioral dyscontrol model of telecommunications fraud.

The retrospective accounts were analytically integrated into a 3-stage interpretive reconstruction—Credulity Priming, Affective Manipulation, and Behavioral Dyscontrol—of how participants described movement from initial engagement to sustained compliance and loss (Figure 2). Categories were assigned to stages according to their function in the narrated fraud process. The resulting framework links trust opening, affective escalation, and behavioral compliance but should not be read as a real-time map of participants’ cognitive or affective states during fraud exposure.

##### Credulity Priming

Credulity Priming functions as the entry stage of the fraud process. Here, scammers create a plausible point of contact by combining communication channels, impersonated identities, and tailored fraud scenarios. The interview data showed that defrauded people were often approached through highly ordinary and familiar routes, such as “My QQ account received a message from my classmate’s QQ,” “a WeChat account suddenly added me,” or “I was at home watching TV and received a call from an unknown number.” At the same time, scammers strengthened plausibility by assuming socially credible identities, for example, “the person claimed to be JD.com customer service,” “claimed to be a police officer from the Public Security Bureau.” Participants’ accounts described these contact channels and fabricated identities as jointly making initial engagement appear credible and situating subsequent events within a scammer-defined interpretive frame. Fraud scenarios then gave this false contact a concrete purpose. In some cases, the opening was linked to anticipated gain or need fulfillment, such as “She said she had tasks to complete and asked me to help then I could take the reward.” In other cases, it was tied to threat and obligation, such as “my daughter must transfer money, or her parents would go to jail” or “my bank card was used for money laundering and I might be a suspect.” What unites these diverse scripts is their function: they make continued engagement appear plausible, justified, and difficult to dismiss.

##### Affective Manipulation

In participants’ retrospective accounts, Affective Manipulation described escalation beyond initial credibility, as scammers used urgency, rewards, threats, and controlled communication channels to intensify emotional involvement. This stage often involves shifting the interaction into scammer-controlled channels and supplying the materials needed to sustain compliance. The interview accounts included behaviors such as “I scanned a QR code and downloaded an app,” “I added their QQ number,” and “I added their WeChat,” indicating that the interaction was gradually relocated into more private and controllable spaces. At the same time, scammers intensified commitment through step-by-step guidance, false proof, and small rewards: one defrauded person reported that the scammer “sent a screenshot showing a successful ¥20,000 transfer to me,” while another recalled, “I sent my WeChat receiving QR code; they transferred ¥60.5, ¥390, and ¥365.47 to me.” In participants’ accounts, these small returns were associated with a desire loop characterized by anticipated gain, hope, and the expectation that a larger payoff was one more step away. Participants also described a parallel fear loop in which narratives of account problems or urgent correction accompanied heightened arousal and narrowed appraisal. Thus, transfer-justification scripts were narrated not merely as explanations for payment but as recurring features accompanying urgency, anxiety, commitment, and continued action. In this sense, initial trust and escalating emotion were narrated as mutually reinforcing influences on continued compliance.

##### Behavioral Dyscontrol

Behavioral Dyscontrol described accounts in which emotional and cognitive pressure accompanied escalating compliance and reduced behavioral flexibility. In this stage, participants no longer simply believed the scammer’s narrative but began to act within it in ways that produce escalating loss. The open-coding material captured this clearly. Participants reported that “after taking a WeLiDai loan I moved ¥5500 into WeChat balance then withdrew to my Postal Bank,” “they told me to transfer money from WeChat/ICBC/BOC/Alipay to my Minsheng Bank, then to their account,” and even “they logged into my Kuaishou via the code I sent and placed two pending orders … I paid them.” These accounts suggest that the final stage is not defined by a single transfer event alone, but by a broader loss of behavioral flexibility under emotional and cognitive pressure. Participants’ accounts described continued compliance even after contradictions began to emerge.

A particularly important dynamic in this model is the role of overexpectation events. In many accounts, scammers first led defrauded people to expect a reward, benefit, or successful resolution, and then suddenly introduced a problem that required further action. For example, defrauded people were told that “the loan was frozen and I had to transfer to a designated account to unfreeze,” or that “only after three more transfers would earlier money be returned.” Others were informed that “the 4th task failed and I must repay ¥7300; then ‘reset tasks,’ continue recharging.” These moments marked a shift from anticipated gain to threatened loss. The defrauded person was no longer acting only to obtain a reward but also to avoid losing previous investments or worsening an already urgent situation. In theoretical terms, overexpectation events were narrated as turning points associated with a shift from anticipated gain to threatened loss. In participants’ accounts, continued compliance was associated not only with hope of gain but also with fear of irreversible damage.

### Theoretical Saturation Check

The first 46 interviews were used for category generation and model development. The remaining 25 interviews were then examined sequentially against the evolving codebook to assess theoretical saturation. During this phase, we specifically evaluated whether any new first-order concepts, higher-order categories, stage assignments, or intercategory relationships emerged that would require the modification of the developing framework. Because the additional 25 interviews did not generate new core categories, did not alter the boundaries of the existing categories, and did not change the proposed stage structure, we judged that theoretical saturation had been achieved.

### Study 2: Quantitative Findings

#### Sample Characteristics

The final analytic sample included 4722 valid participants in total (comprising university students and staff; mean age 20.31, SD 2.862, range 17‐55 y), including 4598 university students and 124 staff members. Among these participants, 409 constituted the confirmed valid sample of participants who had experienced telecommunications fraud, and all were university students. Within this sample (n=409), the average age was 20.03 (SD 1.54, range 17‐25) years, indicating that the defrauded people were exclusively young university students. The sample of participants with confirmed fraud experience comprised 89 (21.8%) male participants and 320 (78.2%) female participants; 255 (62.3%) participants lived in rural areas and 154 (37.7%) in urban areas. For self-rated health, 40 (9.8%) reported very good, 117 (28.6%) good, 117 (28.6%) fair, 125 (30.6%) average, and 10 (2.4%) poor. Family economic status was most often rated average (n=272, 66.5%), followed by relatively poor (n=113, 27.6%), relatively good (n=20, 4.9%), and very good (n=4, 1.0%). Regarding the type of fraud experienced, the most common were impersonation fraud (n=135, 33.0%) and online order-brushing rebate fraud (n=90, 22.0%), followed by fraudulent online game item transactions (n=63, 15.4%) and other forms (n=73, 17.8%); less frequent were lottery, recharge, or gambling fraud (n=17, 4.2%), fraudulent online loans (n=14, 3.4%), fraudulent investment or wealth management (n=12, 2.9%), and online dating or romance fraud (n=5, 1.2%). Reporting behavior showed that 112 (27.4%) participants reported to the police, whereas 297 (72.6%) did not. Among nonreporters, the principal reasons were small financial loss (n=162, 39.6%), other reasons (n=61, 14.9%), lack of evidence (n=40, 9.8%), belief that police could not help (n=27, 6.6%), and embarrassment (n=7, 1.7%).

#### Common Method Bias

For study 2, because all data were collected via self-report, the Harman single-factor test was conducted to assess potential common method bias. An unrotated principal components analysis was performed on all questionnaire items, and the variance explained by the first factor was examined. The first factor accounted for 22.085% of the total variance, which is below the commonly used 40% threshold for severe single-factor dominance. This result suggests that common method variance may not be dominated by a single general factor in the present data.

#### Balanced Case-Control Analysis

To address class imbalance, we constructed a balanced case-control dataset by randomly selecting 409 participants without reported fraud experience from the pool of 4313 such respondents, yielding a 1:1 comparison sample of 818 participants.

**Figure 3.**
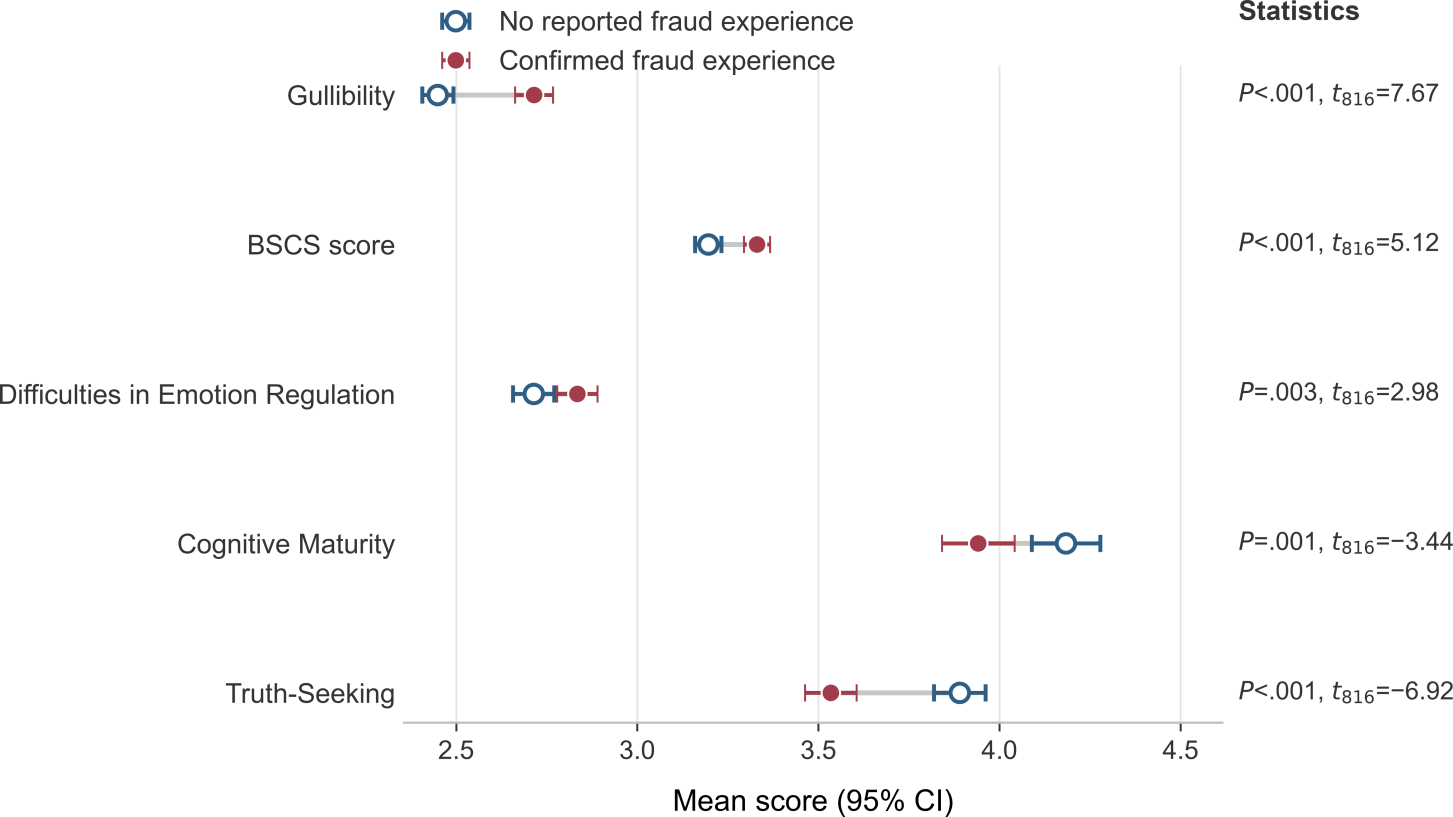
Descriptive group differences between students with confirmed fraud experience and sampled participants without reported fraud experience in the balanced case-control sample. Points indicate group means; horizontal bars indicate 95% CIs. Higher Brief Self-Control Scale (BSCS) scores indicate poorer self-control.

Two-tailed independent-samples *t* tests showed descriptive group differences across the 5 prespecified psychological measures in the balanced case-control dataset. Compared with sampled participants without reported fraud experience, participants with confirmed fraud experience had lower Truth-Seeking and Cognitive Maturity and higher Gullibility, Difficulties in Emotion Regulation, and BSCS scores (higher BSCS scores indicate poorer self-control; all *P*≤.003; Figure 3). These unadjusted contrasts describe group differences and do not imply that all 5 variables independently distinguished victimization status.

#### Logistic Regression

**Figure 4.**
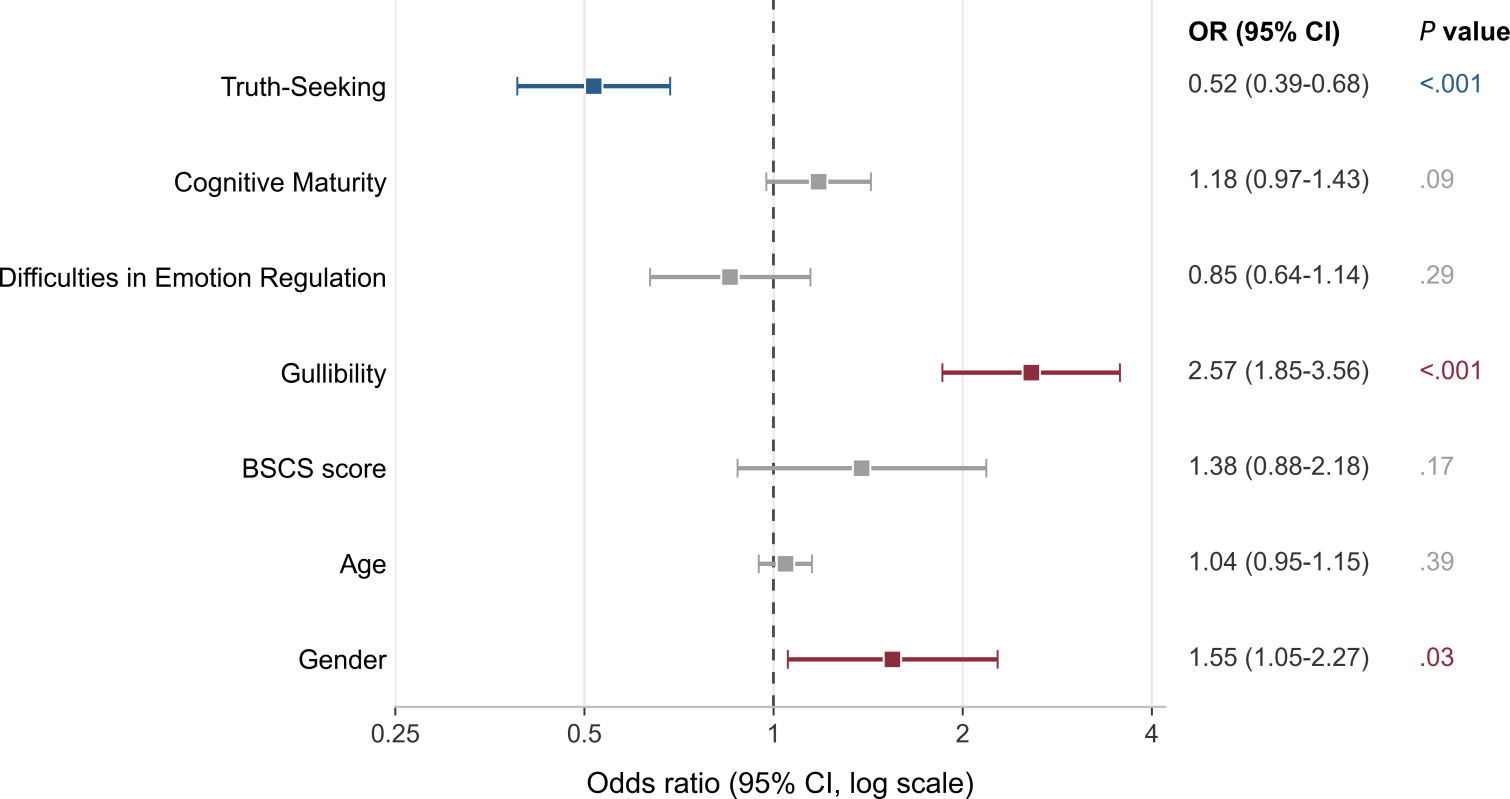
Adjusted odds ratios from the logistic regression model of fraud victimization status in the balanced case-control sample. Higher Brief Self-Control Scale (BSCS) scores indicate poorer self-control. Odds ratios (ORs) were inverted from the original SPSS Exp(B) values so that an OR greater than 1 indicates higher odds of fraud victimization and an OR less than 1 indicates lower odds.

A binary logistic regression examined independent correlates of victimization. The model was significant (omnibus *χ*^2^_7_=95.475; *P*<.001) and showed acceptable calibration (Hosmer-Lemeshow *χ*^2^_8_=13.357; *P*=.10). Higher Gullibility was associated with higher odds of victimization (OR 2.57, 95% CI 1.85‐3.56; *P*<.001), whereas higher Truth-Seeking was associated with lower odds (OR 0.52, 95% CI 0.39‐0.68; *P*<.001). Cognitive Maturity, Difficulties in Emotion Regulation, and BSCS scores were not significant in the adjusted model (Figure 4).

#### Student-Only Sensitivity and Resampling Analyses

The 10-iteration full-pool resampling check and the student-only sensitivity analysis reproduced all descriptive group differences (with the largest observed *P* value being .04; Tables S4 and S5 in Multimedia Appendix 1). Because all confirmed defrauded people were students, the interpretation of adjusted correlates was based primarily on the student-only models: Truth-Seeking and Gullibility were the only psychological correlates consistently reproduced, and the full-pool models yielded the same pattern (Tables S6 and S7 in Multimedia Appendix 1).

Accordingly, lower Truth-Seeking and higher Gullibility were treated as the most stable adjusted correlates. Cognitive Maturity, Difficulties in Emotion Regulation, and BSCS scores showed reliable descriptive differences but not consistent independent associations after adjustment for the other psychological variables and covariates.

#### Within-Group Correlations and Theory-Specified Cross-Sectional Indirect Associations Among Participants With Confirmed Fraud Experience

**Figure 5.**
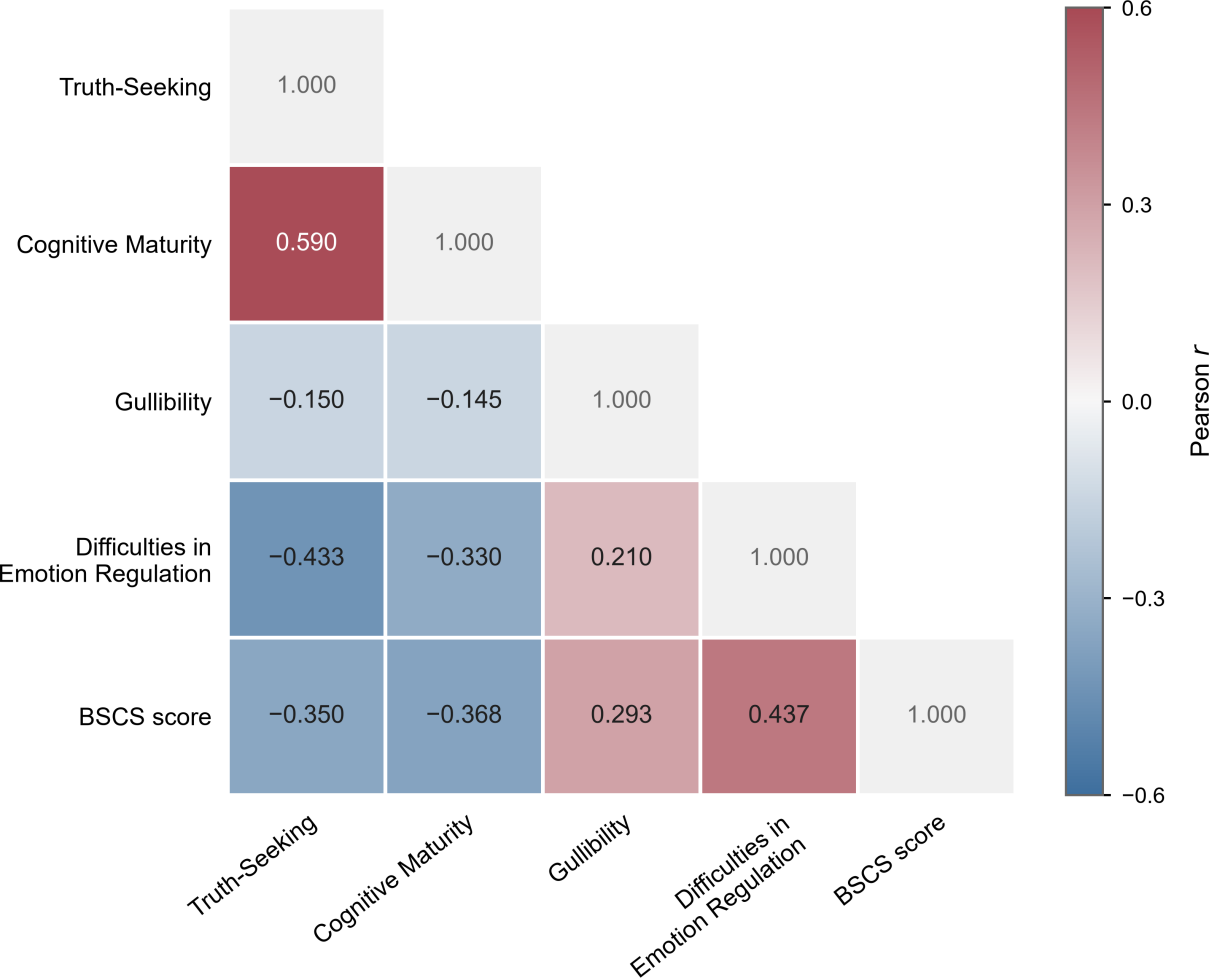
Pearson correlations among study variables within the sample of participants with confirmed fraud experience only (n=409). Higher Brief Self-Control Scale (BSCS) scores indicate poorer self-control.

Figure 5 summarizes within-defrauded people descriptive statistics and correlations (n=409). Truth-Seeking and Cognitive Maturity were positively correlated, and each was inversely correlated with Gullibility, Difficulties in Emotion Regulation, and BSCS score; Gullibility and emotion-regulation difficulties were positively correlated with BSCS scores (higher scores indicate poorer self-control). The correlations between Truth-Seeking and Gullibility and between Cognitive Maturity and Gullibility had *P* values of .002 and .003, respectively; all remaining correlations had *P*<.001. These postevent within-group associations should not be interpreted as case-control predictors of victimization.

#### Theory-Specified Analyses Among Defrauded People

Among defrauded people, we estimated model-specified cross-sectional indirect associations in 2 theory-specified PROCESS Model 6 analyses, with Truth-Seeking and Cognitive Maturity entered separately as focal variables and Gullibility and Difficulties in Emotion Regulation entered as model-specified intermediate variables. Complete regression coefficients are reported in Tables S8 and S9 in Multimedia Appendix 1. Because all measures were collected concurrently after victimization, the estimates are model-specified cross-sectional associations and do not establish temporal order or causality.

**Figure 6.**
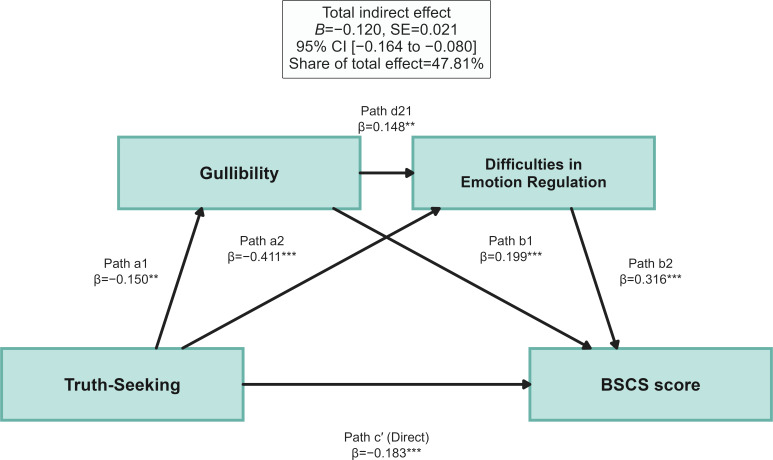
Theory-specified PROCESS Model 6 of cross-sectional indirect associations involving Truth-Seeking, Gullibility, Difficulties in Emotion Regulation, and Brief Self-Control Scale (BSCS) score. The variable arrangement is model-specified and does not depict temporal transmission or causal mediation. Higher BSCS scores indicate poorer self-control. Asterisks indicate *P*<.01 (**) and *P*<.001 (***).

The theory-specified model yielded a total cross-sectional indirect-association estimate for Truth-Seeking and BSCS score (estimate −0.120, SE 0.021, 95% CI −0.164 to −0.080), representing 47.81% of the total association (Table 1). All 3 model-specified component estimates excluded zero, and the direct association remained significant (estimate −0.131, SE 0.034, 95% CI −0.199 to −0.064). Higher Truth-Seeking was associated with better self-control, reflected by lower BSCS scores; the model is shown in Figure 6.

**Table 1.** Theory-specified cross-sectional indirect-association estimates for the Truth-Seeking model^a^.

Model-specified component	Estimate (SE; 95% CI)	Indirect association (%)	Total association (%)
Gullibility	−0.021 (0.009; −0.042 to −0.006)	18	8.37
Difficulties in Emotion Regulation	−0.093 (0.019; −0.133 to −0.058)	78	37.05
Gullibility and Difficulties in Emotion Regulation	−0.005 (0.003; −0.012 to −0.001)	4.17	1.99
Total indirect association	−0.12 (0.021; −0.164 to −0.08)	—^b^	47.81
Direct association	−0.131 (0.034; −0.199 to −0.064)	—	52.19
Total association	−0.251 (0.033; −0.317 to −0.186)	—	100

a Estimates are based on a theory-specified PROCESS Model 6 with 5000 bootstrap resamples in the postevent victim sample (n=409). Component labels identify terms in a cross-sectional decomposition and do not imply temporal transmission or causal mediation.

b Not applicable.

In the Cognitive Maturity model, Cognitive Maturity was negatively associated with Gullibility and Difficulties in Emotion Regulation, whereas Gullibility was positively associated with emotion-regulation difficulties. In the final model, Cognitive Maturity, Gullibility, and emotion-regulation difficulties were each associated with BSCS score. Complete coefficients are reported in Table S9 in Multimedia Appendix 1.

**Figure 7.**
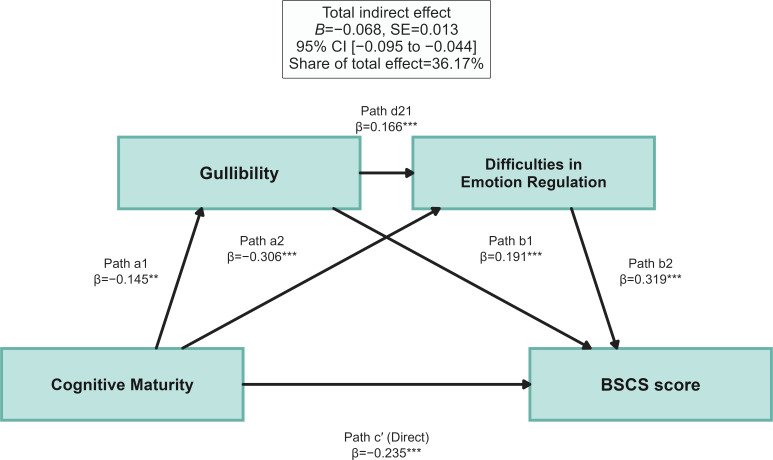
Theory-specified PROCESS Model 6 of cross-sectional indirect associations involving Cognitive Maturity, Gullibility, Difficulties in Emotion Regulation, and Brief Self-Control Scale (BSCS) score. The variable arrangement is model-specified and does not depict temporal transmission or causal mediation. Higher BSCS scores indicate poorer self-control. Asterisks indicate *P*<.01 (**) and *P*<.001 (***).

The theory-specified model yielded a total cross-sectional indirect-association estimate for Cognitive Maturity and BSCS score (estimate −0.068, SE 0.013, 95% CI −0.095 to −0.044), representing 36.17% of the total association (Table 2). All 3 model-specified component estimates excluded zero, and the direct association remained significant (estimate −0.120, SE 0.023, 95% CI −0.165 to −0.075). Higher Cognitive Maturity was associated with better self-control, reflected by lower BSCS scores; the model is shown in Figure 7.

**Table 2.** Theory-specified cross-sectional indirect-association estimates for the Cognitive Maturity model^a^.

Model-specified component	Estimate (SE; 95% CI)	Indirect association (%)	Total association (%)
Gullibility	−0.014 (0.006; −0.027 to −0.004)	21	7.45
Difficulties in Emotion Regulation	−0.05 (0.011; −0.074 to −0.029)	74	26.60
Gullibility and Difficulties in Emotion Regulation	−0.004 (0.002; −0.009 to −0.001)	5.88	2.13
Total indirect association	−0.068 (0.013; −0.095 to −0.044)	—^b^	36.17
Direct association	−0.12 (0.023; −0.165 to −0.075)	—	63.83
Total association	−0.188 (0.024; −0.234 to −0.142)	—	100

a Estimates are based on a theory-specified PROCESS Model 6 with 5000 bootstrap resamples in the postevent victim sample (n=409). Component labels identify terms in a cross-sectional decomposition and do not imply temporal transmission or causal mediation.

b Not applicable.

### Mixed Methods Integration of Findings

Across studies, the clearest convergence concerned trust-related appraisal: study 1 organized credibility construction as the opening domain of defrauded people’s retrospective accounts, whereas student-only sensitivity models of study 2 consistently reproduced lower Truth-Seeking and higher Gullibility as adjusted correlates of victimization status. The phases were complementary across analytic levels, with study 1 providing an event-level interpretation of narrated changes in trust, affect, and behavioral flexibility and study 2 describing conceptually corresponding person-level associations after victimization. Across the remaining domains, Difficulties in Emotion Regulation and BSCS scores showed descriptive group differences or within-defrauded people associations but were not consistently retained as independent correlates in adjusted resampling models. Affective Manipulation and Behavioral Dyscontrol therefore rest primarily on study 1, with study 2 providing complementary quantitative context; Cognitive Maturity likewise lacked stable adjusted case-control support. Thus, the mixed methods findings connected event-level narratives with person-level associative patterns while remaining distinct from claims about stage order or causal relations among constructs.

## Discussion

### Principal Findings

Using a mixed methods design, we proposed a 3-stage interpretive framework of telecommunications fraud—Credulity Priming, Affective Manipulation, and Behavioral Dyscontrol—based primarily on retrospective defrauded people’s narratives in study 1. Importantly, the 2 studies operate at different analytic levels. Study 1 generated an event-level interpretation of how fraud episodes were retrospectively narrated, whereas study 2 examined person-level cross-sectional correlates of victimization status and postevent variation among defrauded people. Together, these findings generate stage-specific prevention hypotheses but provide only cross-sectional quantitative elaboration; prospective evidence is required for temporal or intervention claims.

### Constructing the 3-Stage Model of “Credulity Priming-Affective Manipulation-Behavioral Dyscontrol”

Study 1 organized retrospective accounts into Credulity Priming, Affective Manipulation, and Behavioral Dyscontrol, summarizing the narrated patterns of entry into scammer-crafted scenarios, heightened affect and narrowed appraisal, and escalating compliance. These stages are interpretive categories rather than directly observed psychological transitions.

Our model is theoretically aligned with the dual-process perspective. Rusch [36] argued that the persuasive mechanisms in online fraud primarily operate via 2 routes: a central route and a peripheral route. The central route elicits favorable responses through systematic, logical arguments that prompt deeper elaboration and agreement, whereas the peripheral route relies on ancillary cues and mental shortcuts, bypassing analytic reasoning to secure acceptance without extensive thought. Online scams typically exploit the peripheral route, as they cannot credibly deploy logical arguments to deceive individuals. This tendency is especially pronounced when cognitive resources are limited, leading individuals to depend on peripheral cues rather than the central route [37]. Previous empirical findings are broadly consistent with elements of this dual-process account [38-40]. The qualitative findings extend this general dual-process account by organizing participants’ retrospective descriptions of trust opening, emotional escalation, and behavioral compliance into a staged interpretive sequence. This stage-based specification is theoretically important because future digital public health intervention studies require not only knowledge of which bias exists but hypotheses about when that bias may become most actionable in a live digital encounter [6,8].

The retrospective accounts also highlighted “over-expectation events” as narrated shifts from anticipated benefit to threatened loss. Prior research suggests that managing abrupt emotional reversals may tax emotion-regulation and cognitive-control resources [2,41,42], providing one possible interpretation of these accounts rather than evidence of an observed within-episode mechanism. Similar patterns have been described in romance and order-brushing fraud. These narrated turning points generate the hypothesis that risk may vary across an interaction and could be examined using real-time methods in future just-in-time adaptive intervention research [26].

### Cross-Sectional Quantitative Elaboration

Study 2 provided complementary person-level associations within a predominantly female university-student sample. The interpretation of adjusted correlates prioritized the student-only sensitivity models: lower Truth-Seeking and higher Gullibility were consistently reproduced, whereas Difficulties in Emotion Regulation and BSCS scores appeared mainly as descriptive group differences or within-defrauded people associations. Their relevance to Affective Manipulation and Behavioral Dyscontrol was therefore conceptual rather than independent quantitative support; Cognitive Maturity was likewise not a stable adjusted correlate. The cross-sectional postevent design cannot establish temporal order, distinguish antecedents from consequences, or generalize these patterns beyond the sampled population. These results accord with work linking reasoning and critical thinking to fraud susceptibility and self-regulation [43-48], emotion-related urgency and arousal to risky action [49-53], and dual-process accounts of judgment [54].

Among confirmed students who experienced telecommunications fraud, higher critical-thinking-related scores, lower Gullibility, and better self-control (lower BSCS scores) co-occurred in the theory-specified cross-sectional models. These model-specified associations do not establish direction or temporal ordering. In participants’ accounts, tactics such as urgent transfers, large rewards, or forged official identities accompanied pressured or insufficiently scrutinized choices. Prior research likewise associates higher Gullibility with lower scrutiny of unreliable claims and higher critical thinking with better misinformation discrimination [33,55]. This associative pattern gives the Credulity Priming stage potential translational relevance by suggesting that trust calibration, source verification, and resistance to unverified urgency cues warrant evaluation as candidate early-stage intervention targets.

Within these boundaries, the combined findings generate hypotheses about trust calibration, affective arousal, and self-regulatory difficulty around fraud experiences. Prospective, simulated, or platform-based studies should test whether verification prompts, emotion-sensitive interruption, transaction-level friction, or postvictimization support improve verification, reduce compliance, or aid recovery. Until then, these approaches remain design hypotheses rather than intervention recommendations.

### Limitations and Future Directions

First, the qualitative data for study 1 were obtained through collaboration with a single Public Security Bureau in Chongqing. Although police-verified contact lists improved the authenticity, this localized sampling may introduce geographic and jurisdictional bias. Fraud scripts, emotional triggers, and sociocultural vulnerabilities may vary across regions and countries [56]. In addition, the retrospective interviews may be affected by recall bias and postevent rationalization, especially regarding defrauded people’s real-time emotional states and cognitive processes. Future cross-regional and cross-cultural studies, as well as real-time methods such as ecological momentary assessment, could further test the applicability of the 3-stage framework.

Second, study 2 was conducted in 1 Chongqing university, and all confirmed defrauded people were young students, predominantly women (n=320, 78.2%). This demographic and recruitment profile limits external validity; the case-control, robustness, and within-defrauded people findings should not be treated as population estimates. Replication is needed among older adults, nonstudents, and groups with varied socioeconomic and educational backgrounds.

Third, this study focuses heavily on individual-level psychological variables—such as gullibility, emotion regulation, and critical thinking. It provides a relatively limited analysis of situational and contextual factors that may interact with these individual traits. External cues dynamically controlled by the scammer—such as the intensity of the threat, the credibility of the impersonation, or the time pressure imposed—may interact significantly with defrauded people’s individual vulnerabilities [57]. Future studies could use scenario-based experiments to simulate varied fraud contexts and examine person × situation interactions.

Finally, study 2 measured all constructs after victimization and did not observe within-episode transitions or longitudinal recovery. The associations may therefore reflect antecedents, consequences, or reciprocal relationships. This concern is the strongest for Cognitive Maturity, Difficulties in Emotion Regulation, and BSCS scores, which showed descriptive differences but less stable adjusted associations. Longitudinal, real-time, and simulated designs are needed to clarify directionality and recovery trajectories.

### Conclusions

This study developed a 3-stage interpretive framework from retrospective defrauded people’s narratives. Quantitative evidence was the strongest for trust-related appraisal, with the student-only sensitivity models consistently reproducing lower Truth-Seeking and higher Gullibility as adjusted correlates of victimization status; the findings for Difficulties in Emotion Regulation and BSCS provided complementary context for Affective Manipulation and Behavioral Dyscontrol. The framework offers hypotheses for prospective prevention research; its temporal sequence, broader generalizability, and intervention applications require direct testing.

## Supplementary material

10.2196/87395 Multimedia Appendix 1 Supplementary tables for data analysis.

10.2196/87395 Checklist 1 COREQ checklist for qualitative research.

10.2196/87395 Checklist 2 CHERRIES checklist for the online survey.

## Abbreviations

BSCS: Brief Self-Control Scale
CHERRIES: Checklist for Reporting Results of Internet E-Surveys
COREQ: Consolidated Criteria for Reporting Qualitative Research
CTDI–CV: Chinese version of the Critical Thinking Disposition Inventory
DERS: Difficulties in Emotion Regulation Scale
