# Supplementary material for: Psychological Processes of Telecommunications Fraud: Development of a Three-Stage Framework and Cross-Sectional Quantitative Elaboration in a Mixed Methods Study
Source: J Med Internet Res. 2026 Aug 13;28:e87395. doi: 10.2196/87395 (PMC13472532; doi:10.2196/87395)
Supplement: Supplementary file 1 [file jmir-v28-e87395-s001.docx]

### Open coding

Open coding constituted the first stage of data analysis (Appendix Table A1). In this phase, the transcripts were examined line by line and meaning units related to contact channels, impersonated identities, fraud scenarios, emotional responses, behavioral inducements, and transfer-related actions were labeled. Codes were then compared, merged, revised, and renamed as needed. A total of 71 initial concept nodes and 27 dimensions were identified, providing the descriptive foundation for subsequent axial and selective coding.

Table S1. Illustrative Examples of Open Coding

| **Category** | **Original Material** | **Initial Open Code** |
| --- | --- | --- |
| Social media & communication channels | My QQ account received a message from my classmate’s QQ. | Received QQ message |
|  | While I was in Beijing, a WeChat account suddenly added me. | Added on WeChat |
|  | A stranger added me on Weibo and then said she had tasks to be completed. | Added on Weibo |
|  | To communicate while gaming, my friend (screen name “Yiba Mùjiàn”) and I created a room on YY Voice to chat and play. | YY Voice |
|  | After I logged into DingTalk meetings, the other party sent me a meeting number; I entered it and joined the meeting. | Video meeting |
|  | I downloaded “LeTV Screen Casting” and “Tencent Meeting,” and then we used these two apps to communicate. | Video meeting |
| Non-official app channels | At my home (No. 206 Naxing Road, Unit 4-1), I downloaded the “Wanhe Juejin” app and registered. Then the group owner sent a link to download “Juying Media,” saying tasks would be assigned there. | Downloaded non-official apps via other channels |
| Life-service & e-commerce platforms | I wanted to get my overseas diploma certified, so on Feb 26, 2023 I looked for a third-party service on Xiaohongshu. | Xiaohongshu |
|  | I searched “xyk” (credit card initials) on Xianyu (IdleFish). | IdleFish |
| Information pushes/ads | Last night in the hotel I was bored and browsing online when I came across a webpage. While reading an online novel, a pop-up ad appeared. | Website or pop-up |
|  | I saw a “Chongqing Same-City Online” QR code sticker on my car’s left mirror. | Ad card |
| Traditional communication | I was at home watching TV and received a call from an unknown number. | Phone call |
|  | When I was bored I saw a text message with a link to download a cash-out app. | SMS |
| Impersonated identity – Customer service | At work I received a call; the person claimed to be JD.com customer service. Later they said an ICBC customer service agent would help cancel my membership. | E-commerce/financial customer service |
| Impersonated identity – Non-official app staff | A contact named “Jiali Consultant–Jingjing” reached out through the app; on the “Bailian” app I added two “guides.” | Non-official app customer service |
| Impersonated identity – Short-video app CS | I received a call claiming to be Douyin customer service; another claimed to be Kuaishou customer service. | Short-video app CS |
| Impersonated identity – Acquaintance/family | I got a message on Weibo from a friend. | Friend |
|  | A YY account with the same nickname and avatar as my online friend “Chenyong” pulled me into a private discussion group. | Online friend |
|  | Someone added me on QQ saying he was my son. | Family member |
| Impersonated identity – Business contact | The person said they sold “Chi essential oil”; I sell facial masks. Someone added me on WeChat to buy goods. | Peer trader |
|  | At noon, an officer assigned me to buy supplies from a person named Wang Anbao. | Supplier |
| Impersonated authority | Claimed to be Li, a squad leader from the Armed Police in Liangshan Prefecture, Sichuan. | Armed police |
|  | Claimed to be a police officer from the Public Security Bureau. | Police |
|  | Someone added me on WeChat, saying he was in the army. | Soldier |
| Fraud scenario – Financial services | I needed cash flow and saw the Changxiangjin app on Bajie Finance. I successfully applied for a ¥20,000 loan; it showed as balance but required a “withdrawal code” from customer service. | Providing loans |
|  | They said my JD BaiTiao interest rate was too high and would help adjust it and delete the record. | Interest-rate adjustment |
|  | My credit card was due and I wanted to cash out with another card… He charged ¥10 per ¥1,000. He needed to place orders via Kuaishou on my account. | Credit-card cash-out |
|  | They told me to close JD JinTiao and BaiTiao. I couldn’t; they said they’d help operate WeBank’s WeLiDai. | Closing financial services |
| Fraud scenario – Fake goods/services | They said my Douyin e-commerce membership had been opened; I didn’t know about it. They told me to cancel it. | Cancel paid service |
|  | I could use the website to refund the tuition previously paid for self-study undergrad. | Fee refund |
|  | In a gaming group someone said he was moving back to Beijing and had a desktop set to give away, but I’d need to pick it up after delivery. | Free item |
|  | They proposed cross-promoting each other’s products, but said my “micro-business level” was too low and I had to upgrade to sell to them; or buy via designated suppliers. | Item purchase requirement |
| Fraud scenario – Need fulfillment | She said she had tasks to complete—“order brushing”—and asked me to help; I could keep the income. | Order-brushing rebates |
|  | An app contact “Jiali Consultant–Jingjing” said the platform was for online hook-ups. | Casual sex |
|  | They said they could arrange on-site prostitution. | Prostitution |
|  | I saw matchmaking info on WeChat; they said I needed to become a member. | Matchmaking |
| Emotional manipulation | In the group they said my daughter must transfer money, or her parents would go to jail. | Threatening family safety |
|  | Claimed my bank card was used for money laundering and I might be a suspect, so I must cooperate with police by transferring money to a designated account. | Alleged criminal involvement |
|  | On QQ they said his cousin was in surgery and urgently needed money; another with the same nickname/avatar as my friend privately asked me to borrow money for him; my “son” said there was an elite Tsinghua training program requiring a fee. | Impersonated acquaintance/family asking for help |
| Inducing behavior – Provide URLs | They asked me to search “授权信息注销.com” in the browser. | Provided website |
| Inducing behavior – Provide links | I did order brushing by following her link. | Provided link |
| Inducing behavior – Provide contact info | He pushed me a QQ number. | Social-media handle provided |
|  | He gave me the manufacturer’s phone number. | Phone number provided |
| Inducing behavior – Provide payment method | Sent me a WeChat Pay QR code. | QR code provided |
|  | Gave me a bank account number. | Bank account provided |
| Inducing behavior – Fake proofs | Sent a screenshot showing a successful ¥20,000 transfer to me. | Fake transfer screenshot |
|  | Sent a red-headed official document labeled “Chinese People’s Armed…” | Fake official document |
| Small rebates | I sent my WeChat receiving QR code; they transferred ¥60.5, ¥390, and ¥365.47 to me. | Scammer rebates to victim |
| Victim behavior – Fund transfer & payment | After taking a WeLiDai loan I moved ¥5,500 into WeChat balance then withdrew to my Postal Bank. | Loan and transfer |
|  | They told me to transfer money from WeChat/ICBC/BOC/Alipay to my Minsheng Bank, then to their account. | Bank transfer |
|  | They logged into my Kuaishou via the code I sent and placed two pending orders for virtual recharge cards (¥2,000 & ¥5,000). I paid them. | Paying orders |
| Victim behavior – Adding scammer contacts | I scanned a QR code and downloaded an app. | Downloaded app |
|  | I added their QQ number. | Added scammer’s QQ |
|  | I added their WeChat. | Added scammer’s WeChat |
| Transfer-justification scripts – Account freeze | CS said the bank number I entered was wrong; the loan was frozen and I had to transfer to a designated account to unfreeze. | Funds frozen; transfer to unfreeze |
|  | They said a guarantee was needed for the money I just transferred, requiring another ¥60,000. | Transfer as guarantee |
|  | They said their WeChat/Alipay were restricted; I needed to transfer money to lift the freeze, after which they’d return ¥1,500 deposit. | Payment functions restricted; transfer to unfreeze |
| Transfer-justification scripts – Criminal suspicion/record | Claimed I might be involved in a crime and must cooperate with law enforcement by putting funds in a designated account. | Transfer to “cooperate with law enforcement” |
|  | Told me to take a WeLiDai loan and transfer to a specified account to “remove bad records.” | Loan then transfer to clear record |
| Transfer-justification scripts – Operational error/insufficient steps | After a while they told me to keep transferring—only after three more transfers would earlier money be returned. | Keep transferring to recover funds |
|  | App CS said the 4th task failed and I must “补单” ¥7,300; then “reset tasks,” continue recharging. | Operation error—must continue |
| Transfer-justification scripts – Operate financial products | They said all my bank cards had loan limits; I must draw them out and transfer to a “verification account,” then they’d refund and close JD JinTiao. | Transfer to cancel financial products |
|  | If I emptied my bank/online-loan limits and transferred to the credit center for verification, I’d qualify for lower interest. | Loan then transfer to lower rate |
| Transfer-justification scripts – Advance purchase/cover for others | Asked me to transfer money to buy a bag / help relatives place orders / pay hospital deposit first (¥3,000) because bank upgrade delayed. | Advance payment for others |
| Transfer-justification scripts – Order brushing/investment | People were doing order brushing for rewards; I followed to get rewards. | Followed others to brush orders |
|  | “Jiali Consultant–Jingjing” said it’s a hook-up platform; must register as member and finish 4 tasks first. | Brush orders to hook up |
|  | A stock group promised a “special channel”; recommended stocks seemed to hit limit-up; I followed. | Followed others to trade stocks |
| Transfer-justification scripts – Must transfer to transact | The supplier insisted on private transfer instead of corporate remittance to ship goods. | Pay first to purchase |
|  | I had to pay to upgrade my reseller level before they’d buy from me; after one transfer they kept asking for more for new “levels.” | Transfer required to meet transaction preconditions. |

### Axial coding

Axial coding yielded six primary categories, revealing a progressive logic of trust formation → affective arousal → behavioral inducement in telecommunications fraud operations (Table A2). First, under communication channels, scammers typically establish initial contact through multiple routes—online social platforms, information-push mechanisms, and traditional telecommunication—to initiate outreach. Second, impersonated identities constitute a key manipulation strategy, with scammers posing as customer service representatives, acquaintances/family, business partners, or authority figures to reduce victims’ vigilance. Third, fraud scenarios are constructed to activate emotion and trust, using scripted situations of “need-fulfillment” or “emotional manipulation,” thereby transitioning victims into the action stage. Subsequent behavioral inducements include providing contact details, steering payment/transfer methods, prompting fund transfers, offering small “rebates,” and supplying forged proofs or fabricated “evidence,” all of which deepen psychological engagement. Finally, with respect to transfer-justification scripts, the codes show that actions are frequently justified by plausibilizing narratives crafted by scammers—such as “account frozen—unfreezing required,” “record removal or law-enforcement cooperation,” “remedying operational errors,” or “investment/order-brushing tasks”—which further dull victims’ sensitivity to anomalous requests.

Table S2. Axial Coding

| **Primary Category** | **Secondary Category** |
| --- | --- |
| Communication channels | Online social-platform contact channels |
|  | Information push / advertising channels |
|  | Traditional telecommunication channels |
| Impersonated identities | Customer service representative |
|  | Acquaintance or family member |
|  | Business/event counterpart |
|  | Authority figure (official/agency) |
|  | Financial-service personnel |
|  | Fake stores and service providers |
| Fraud scenarios | Need-fulfillment scripts (help, benefits, opportunities) |
|  | threat- or obligation-based scripts |
|  | Opportunity- or reward-based scripts |
| Scammer behaviors | Providing contact information |
|  | Providing payment or transfer methods |
|  | Small “rebate/return” incentives |
|  | Supplying forged proofs and fabricated “evidence” |
| Victim behaviors | Migrating to scammer-controlled contact channels |
|  | Transferring funds / making payments |
| Transfer-justification scripts | “Account or funds are frozen; you must unfreeze the account.” |
|  | “You are suspected of a crime or have a negative record; the account must cooperate with verification or ‘record removal.’” |
|  | “An operation error/insufficiency occurred; you must keep operating with the account to resolve it.” |
|  | “The account is needed to operate financial products.” |
|  | “Please purchase on someone’s behalf / make an advance payment.” |
|  | “The account is required for order-brushing / ‘investment tasks.’” |
|  | “The account is required to complete the transaction.” |

### Selective Coding

Building on the axial coding, the results of selective coding were integrated into 3 sequential stages, yielding the final selective-coding schema shown in Table A3. At this stage of analysis, the purpose was no longer simply to list categories, but to integrate them into a coherent explanatory structure. Following grounded-theory logic, the categories were selectively organized according to their functional role in the unfolding fraud trajectory, thereby forming an interrelated storyline rather than a set of parallel themes. In this model, telecommunications fraud was conceptualized as a staged process in which scammers first open a pathway for misplaced trust, then intensify emotional involvement and urgency, and finally convert this altered cognitive-affective state into concrete compliance behaviors.

Table A3 therefore reflects the transition from descriptive categorization to theoretical integration. Categories assigned to Credulity Priming were those that primarily functioned to establish plausibility, lower vigilance, and draw the victim into a scammer-defined interpretive frame. These included communication channels, impersonated identities, and fraud scenarios. Categories assigned to Affective Manipulation were those that functioned to escalate commitment, maintain emotional arousal, and make compliance seem necessary or urgent. These included scammer behaviors such as providing contact information, payment routes, rebates, and fabricated evidence, as well as transfer-justification scripts that framed continued action as reasonable, necessary, or unavoidable. Categories assigned to Behavioral Dyscontrol were those that reflected the victim’s enacted compliance under emotional and cognitive pressure, especially migration into scammer-controlled contact channels and the performance of actual payments or transfers.

Importantly, the stage assignments were based on functional role rather than surface wording alone. For example, “transfer-justification scripts” were retained in the Affective Manipulation stage because they did not merely explain transfers descriptively; rather, they served as narrative devices through which urgency, fear, anticipated recovery, or promised gain were sustained and translated into action. Likewise, “migrating to scammer-controlled contact channels” was retained under Behavioral Dyscontrol because, in the present data, this behavior often represented a concrete step of compliance that deepened scammer control and reduced victims’ opportunity for independent verification. Thus, the selective-coding schema should be understood as a theoretically integrated map of the fraud process, rather than as a simple classification of isolated content categories.

Table S3. Selective Coding

| **Stage** | **Primary Category** | **Secondary Category** |
| --- | --- | --- |
| **Credulity Priming** | Communication Channels | Social-media & messaging channels / Unregulated app channels / Lifestyle services & e-commerce platforms / Information-push or advertising channels / Traditional telecommunication channels |
|  | Impersonated Identities | Customer-service representative / Acquaintance or family member / Business counterpart / Authority figure / Financial-service personnel |
|  | Fraud Scenarios | Counterfeit goods & services / Need-fulfillment scripts / Emotional manipulation |
| **Affective Manipulation** | Scammer Behaviors | Providing contact information / Payment or transfer methods / Small “rebate/return” incentives / Forged proofs & fabricated “evidence” |
|  | Transfer-justification scripts | “Account/funds frozen; transfer required to unfreeze.” / “Suspected crime or negative record; transfer required to cooperate or remove record.” / “Operational error; continued transfers needed to resolve.” / “Transfers needed to operate financial products.” / “Advance purchase/payment on behalf of others.” / “Order-brushing or ‘investment tasks’.” / “Transfer required to complete the transaction.” |
| **Behavioral Dyscontrol** | Victim Behaviors | Migrating to scammer-controlled contact channels / Transferring funds or making payments |

Table S4. Results of 10-Iteration Non-Overlapping Resampling Robustness Check (Welch's t tests)

| Iteration | **Truth-Seeking (t)** | **Cognitive Maturity (t)** | **Gullibility (t)** | **Difficulties in Emotion Regulation (t)** | **BSCS score (t)** |
| --- | --- | --- | --- | --- | --- |
| Iteration 1 | -6.32*** | -3.41*** | 7.09*** | 3.59*** | 5.34*** |
| Iteration 2 | -6.27*** | -3.46*** | 7.47*** | 2.36* | 4.67*** |
| Iteration 3 | -6.45*** | -3.31*** | 7.95*** | 3.79*** | 5.25*** |
| Iteration 4 | -6.36*** | -2.92** | 6.66*** | 3.52*** | 4.40*** |
| Iteration 5 | -6.48*** | -2.78** | 6.67*** | 2.60** | 4.72*** |
| Iteration 6 | -6.15*** | -3.25** | 6.47*** | 2.63** | 3.75*** |
| Iteration 7 | -6.73*** | -3.34*** | 7.14*** | 3.27** | 4.55*** |
| Iteration 8 | -6.56*** | -4.12*** | 7.69*** | 3.48*** | 5.27*** |
| Iteration 9 | -6.49*** | -3.09** | 7.09*** | 3.60*** | 5.56*** |
| Iteration 10 | -6.37*** | -3.37*** | 6.54*** | 4.22*** | 4.68*** |

Note. N = 818 for each iteration. (nvictim = 409, nnon-victim = 409). Non-victims were computationally drawn from a larger pool (N = 4,313) using simple random sampling without replacement across iterations (total unique non-victims utilized = 4,090). A negative t-value indicates that victims scored significantly lower than non-victims; a positive t-value indicates that victims scored significantly higher. *p < .05, **p < .01, ***p < .001.

Table S5. Results of 10-Iteration Non-Overlapping Resampling Robustness Check (Welch's t tests, Student-Only Control Pool)

| **Iteration** | **Truth-Seeking (**t) | **Cognitive Maturity (**t) | **Gullibility (**t) | **Difficulties in Emotion Regulation (**t) | BSCS score (t) |
| --- | --- | --- | --- | --- | --- |
| Iteration 1 | -7.82*** | -4.54*** | 7.49*** | 3.45*** | 5.59*** |
| Iteration 2 | -6.22*** | -3.08** | 7.67*** | 3.65*** | 5.05*** |
| Iteration 3 | -4.92*** | -2.29* | 6.30*** | 2.23* | 4.89*** |
| Iteration 4 | -7.05*** | -2.72** | 6.84*** | 3.52*** | 4.67*** |
| Iteration 5 | -5.58*** | -2.93** | 6.50*** | 4.12*** | 3.99*** |
| Iteration 6 | -5.12*** | -2.27* | 6.02*** | 2.06* | 3.81*** |
| Iteration 7 | -7.66*** | -4.60*** | 7.92*** | 3.38*** | 5.29*** |
| Iteration 8 | -7.75*** | -4.26*** | 8.82*** | 4.57*** | 6.35*** |
| Iteration 9 | -5.09*** | -2.90** | 5.85*** | 2.74** | 3.89*** |
| Iteration 10 | -7.17*** | -3.93*** | 7.96*** | 4.39*** | 5.86*** |

Note. N = 818 for each iteration (nvictim = 409, nnon-victim = 409). To improve comparability between cases and controls, staff respondents were excluded and non-victims were drawn from a student-only pool (N = 4,189) using simple random sampling without replacement across iterations (total unique non-victims utilized = 4,090; remaining unused = 99). A negative t value indicates that victims scored significantly lower than non-victims, whereas a positive t value indicates that victims scored significantly higher than non-victims.
*p < .05, **p < .01, ***p < .001.

Table S6. Results of 10-Iteration Non-Overlapping Resampling Robustness Check (Binary Logistic Regression)

| Iteration | **Gender** | **Age** | **Truth-Seeking** | **Cognitive Maturity** | **Gullibility** | **Difficulties in Emotion Regulation** | BSCS score |
| --- | --- | --- | --- | --- | --- | --- | --- |
| Iteration 1 | 0.81 | 1.09 | 0.59*** | 1.13 | 2.23*** | 1.05 | 1.47 |
| Iteration 2 | 0.92 | 1.08 | 0.60*** | 1.09 | 2.43*** | 0.85 | 1.46 |
| Iteration 3 | 0.87 | 1 | 0.58*** | 1.12 | 2.44*** | 1.02 | 1.45 |
| Iteration 4 | 0.95 | 1.03 | 0.53*** | 1.19 | 2.11*** | 1.05 | 1.16 |
| Iteration 5 | 1.11 | 1.07 | 0.51*** | 1.24* | 2.07*** | 0.91 | 1.54 |
| Iteration 6 | 1.03 | 0.99 | 0.55*** | 1.12 | 2.07*** | 0.91 | 1.13 |
| Iteration 7 | 0.97 | 0.98 | 0.55*** | 1.11 | 2.23*** | 1 | 1.18 |
| Iteration 8 | 0.79 | 1 | 0.59*** | 1.06 | 2.47*** | 0.97 | 1.39 |
| Iteration 9 | 0.92 | 1.1 | 0.55*** | 1.22* | 2.22*** | 1.01 | 1.59* |
| Iteration 10 | 1.07 | 1.05 | 0.58*** | 1.11 | 1.97*** | 1.15 | 1.21 |

Note. N = 818 for each iteration. (nvictim = 409, nnon-victim = 409). Values represent Odds Ratios (OR). Gender is coded with Male as the reference group (Male = 0, Female = 1). Age and Gender were entered as covariates. *p < .05, **p < .01, ***p < .001.

Table S7. Results of 10-Iteration Non-Overlapping Resampling Robustness Check (Binary Logistic Regressions, Student-Only Control Pool)

| **Iteration** | **Gender** | **Age** | **Truth-Seeking** | **Cognitive Maturity** | **Gullibility** | **Difficulties in Emotion Regulation** | BSCS score |
| --- | --- | --- | --- | --- | --- | --- | --- |
| Iteration 1 | 0.86 | 1.05 | 0.53*** | 1.06 | 2.19*** | 0.95 | 1.32 |
| Iteration 2 | 0.88 | 1.05 | 0.61*** | 1.11 | 2.39*** | 1.04 | 1.39 |
| Iteration 3 | 0.66* | 1.04 | 0.64** | 1.15 | 2.11*** | 0.84 | 1.67* |
| Iteration 4 | 1.02 | 1.04 | 0.47*** | 1.30** | 2.14*** | 1.04 | 1.36 |
| Iteration 5 | 1.13 | 1.04 | 0.63*** | 1.15 | 1.95*** | 1.22 | 1.15 |
| Iteration 6 | 0.74 | 0.99 | 0.60*** | 1.13 | 2.11*** | 0.87 | 1.3 |
| Iteration 7 | 1 | 1.09 | 0.52*** | 1.07 | 2.50*** | 1 | 1.29 |
| Iteration 8 | 0.69 | 1.07 | 0.50*** | 1.19 | 2.86*** | 1.06 | 1.57 |
| Iteration 9 | 1.08 | 1.08 | 0.66** | 1.08 | 1.94*** | 0.97 | 1.27 |
| Iteration 10 | 1.12 | 1.09 | 0.54*** | 1.18 | 2.38*** | 1.12 | 1.46 |

Note. N = 818 for each iteration (nvictim = 409, nnon-victim = 409). Values represent odds ratios (ORs). To improve comparability between cases and controls, staff respondents were excluded and non-victims were drawn from a student-only pool (N = 4,189) using simple random sampling without replacement across iterations (total unique non-victims utilized = 4,090; remaining unused = 99). Gender is coded with male as the reference group (male = 0, female = 1). Age and gender were entered as covariates in all models. ORs below 1 indicate lower odds of victimization, whereas ORs above 1 indicate higher odds of victimization.
*p < .05, **p < .01, ***p < .001.

Table S8. Regression Analysis of Truth-Seeking, Gullibility, Difficulties in Emotion Regulation, and the BSCS score

| **Outcome variable** | **Predictor variable** | ***β*** | ***SE*** | ***t*** | ***p*** | ***R*** | ***R²*** | ***F*** |
| --- | --- | --- | --- | --- | --- | --- | --- | --- |
| Gullibility | Truth-Seeking | –0.15 | 0.036 | –3.062 | 0.002 | 0.15 | 0.023 | 9.375^**^ |
| Difficulties in Emotion Regulation | Truth-Seeking | –0.411 | 0.032 | –9.206 | ＜0.001 | 0.457 | 0.209 | 53.698^***^ |
|  | Gullibility | 0.148 | 0.043 | 3.323 | 0.001 |  |  |  |
| BSCS score | Truth-Seeking | –0.35 | 0.033 | –7.538 | ＜0.001 | 0.35 | 0.123 | 56.822^***^ |
| BSCS score | Truth-Seeking | –0.183 | 0.034 | –3.858 | ＜0.001 | 0.510 | 0.260 | 47.535^***^ |
|  | Gullibility | 0.199 | 0.043 | 4.538 | ＜0.001 |  |  |  |
|  | Difficulties in Emotion Regulation | 0.316 | 0.048 | 6.574 | ＜0.001 |  |  |  |

Note. **p < 0.01, ***p < 0.001. BSCS denotes the Brief Self-Control Scale; higher scores indicate poorer self-control.

Table S9. Regression Analysis of Cognitive Maturity, Gullibility, Difficulties in Emotion Regulation, and the BSCS score

| **Outcome variable** | **Predictor variable** | ***β*** | ***SE*** | ***t*** | ***p*** | ***R*** | ***R²*** | ***F*** |
| --- | --- | --- | --- | --- | --- | --- | --- | --- |
| Gullibility | Cognitive Maturity | –0.145 | 0.026 | –2.960 | 0.003 | 0.114 | 0.021 | 8.761^**^ |
| Difficulties in Emotion Regulation | Cognitive Maturity | –0.306 | 0.024 | –6.557 | ＜0.001 | 0.368 | 0.136 | 31.851^***^ |
|  | Gullibility | 0.166 | 0.045 | 3.551 | ＜0.001 |  |  |  |
| BSCS score | Cognitive Maturity | –0.368 | 0.024 | –7.89 | ＜0.001 | 0.368 | 0.135 | 63.686^***^ |
| BSCS score | Cognitive Maturity | –0.235 | 0.023 | –5.244 | ＜0.001 | 0.531 | 0.282 | 53.020^***^ |
|  | Gullibility | 0.191 | 0.042 | 4.431 | ＜0.001 |  |  |  |
|  | Difficulties in Emotion Regulation | 0.319 | 0.046 | 7.054 | ＜0.001 |  |  |  |

Note. **p < 0.01, ***p < 0.001. BSCS denotes the Brief Self-Control Scale; higher scores indicate poorer self-control.

### Appendix S10. Core Domains and Administration of the Semistructured Interview Guide for Study 1

**Administration and Scope**

Interviews followed the event timeline. Participants were first invited to provide an account with minimal interruption, after which the interviewer selected nonmandatory probes relevant to the reported experience. Questions were not required to follow a fixed order, and interviewers were instructed not to impose theoretical stage labels, technical terms, or value judgments on participants' accounts.

**Core Interview Questions**

**Q1.** Please describe, in your own words, how the fraud episode unfolded.

**Q2.** What did the other party say or show when they first contacted you?

**Q3.** What information or details made you willing to continue listening or communicating?

**Q4.** How did the other party guide you, step by step, to continue cooperating?

**Q5.** What were you mainly thinking during the episode?

**Q6.** How did your emotions or physical sensations change as the episode developed?

**Q7.** Was there a key moment when the situation or your feelings changed suddenly?

**Q8.** In chronological order, what actions did you take?

**Q9.** Did you become suspicious or want to stop at any point?

**Q10.** How did the fraud episode end?

**Q11.** Looking back, at what point might the episode most plausibly have been interrupted?

**Q12.** What effects did the experience have on you afterward?

**Q13.** Is there anything important about the experience that we have not asked?

**Q14.** What would you most want to tell others to help prevent similar fraud?

**Optional Probes**

Optional probes were selected in response to each account and addressed perceived credibility and identity cues; verification attempts, time pressure, and expected consequences; attention and cognitive load; emotional and bodily changes; shifts from anticipated gain to threatened loss; transfers and other compliance behaviors; suspicion and difficulty stopping; external intervention; and postevent effects and support.

**Postinterview Field Log**

The guide included a field log for recording new concepts or expressions; evidence consistent with the evolving categories; counterexamples or contradictory material; questions requiring follow-up in subsequent interviews; and potential interviewer assumptions, emotional responses, or interpretive tendencies.
