# Supplementary material for: Psychological Processes of Telecommunications Fraud: Development of a Three-Stage Framework and Cross-Sectional Quantitative Elaboration in a Mixed Methods Study
Source: J Med Internet Res. 2026 Aug 13;28:e87395. doi: 10.2196/87395 (PMC13472532; doi:10.2196/87395)
Supplement: Supplementary file 2 [file jmir-v28-e87395-s002.docx]

| **No** | **Item** | **Reported on Section** |
| --- | --- | --- |
| Domain 1: Research team and reflexivity |  |  |
| 1 | Interviewer/facilitator | Section: Study 1 - Participants |
| 2 | Credentials | Section: Study 1 - Participants |
| 3 | Occupation | Section: Study 1 - Participants |
| 4 | Gender | N/A |
| 5 | Experience and training | Section: Study 1 - Participants |
| 6 | Relationship established | N/A (No prior relationship) |
| 7 | Participant knowledge of the interviewer | Section: Study 1 - Participants |
| 8 | Interviewer characteristics | N/A |
| Domain 2: study design |  |  |
| 9 | Methodological orientation and Theory | Section: Introduction / Study 1 - Data analysis (Grounded theory) |
| 10 | Sampling | Section: Study 1 - Participants |
| 11 | Method of approach | Section: Study 1 - Participants (Telephone) |
| 12 | Sample size | Section: Study 1 - Participants (n=71) |
| 13 | Non-participation | Section: Study 1 - Participants |
| 14 | Setting of data collection | Section: Study 1 - Participants |
| 15 | Presence of non-participants | N/A |
| 16 | Description of sample | Section: Study 1 - Participants |
| 17 | Interview guide | Section: Study 1 - Participants (Semi-structured) |
| 18 | Repeat interviews | N/A |
| 19 | Audio/visual recording | Section: Study 1 - Participants |
| 20 | Field notes | N/A |
| 21 | Duration | Section: Study 1 - Participants |
| 22 | Data saturation | Section: Study 1 - Theoretical Saturation Check |
| 23 | Transcripts returned | N/A |
| Domain 3: analysis and findings |  |  |
| 24 | Number of data coders | Section: Study 1 - Data analysis |
| 25 | Description of the coding tree | Section: Study 1 - Results (Tables 1, 2, 3) |
| 26 | Derivation of themes | Section: Study 1 - Data analysis |
| 27 | Software | Section: Study 1 - Data analysis (NVivo 20) |
| 28 | Participant checking | N/A |
| 29 | Quotations presented | Section: Study 1 - Results (Table 1) |
| 30 | Data and findings consistent | Yes, across all Results sections |
| 31 | Clarity of major themes | Section: Study 1 - Results |
| 32 | Clarity of minor themes | Section: Study 1 - Results |
