# Supplementary material for: Psychological Processes of Telecommunications Fraud: Development of a Three-Stage Framework and Cross-Sectional Quantitative Elaboration in a Mixed Methods Study
Source: J Med Internet Res. 2026 Aug 13;28:e87395. doi: 10.2196/87395 (PMC13472532; doi:10.2196/87395)
Supplement: Supplementary file 3 [file jmir-v28-e87395-s003.docx]

| **Item Category** | **Checklist Item** | **Reported on Section** |
| --- | --- | --- |
| Design | Describe survey design | Section: Study 2 - Participants |
| IRB approval and informed consent process | IRB approval | Section: Ethical Considerations |
|  | Informed consent | Section: Ethical Considerations |
|  | Data protection | Section: Ethical Considerations |
| Development and pre-testing | Development and testing | N/A |
| Recruitment process and description of the sample... | Open survey versus closed survey | Section: Study 2 - Participants |
|  | Contact mode | Section: Study 2 - Participants |
|  | Advertising the survey | Section: Study 2 - Participants |
| Survey administration | Web/E-mail | Section: Study 2 - Participants |
|  | Context | Section: Study 2 - Participants |
|  | Mandatory/voluntary | Section: Ethical Considerations |
|  | Incentives | Section: Ethical Considerations |
|  | Time/Date | Section: Study 2 - Participants |
|  | Randomization of items | N/A |
|  | Adaptive questioning | N/A |
|  | Number of Items | Section: Study 2 - Materials |
|  | Number of screens (pages) | N/A |
|  | Completeness check | Section: Study 2 - Participants |
|  | Review step | N/A |
| Response rates | Unique site visitor | N/A |
|  | View rate | N/A |
|  | Participation rate | Section: Study 2 - Participants |
|  | Completion rate | Section: Study 2 - Participants |
| Preventing multiple entries from the same individual | Cookies used | N/A |
|  | IP check | Section: Study 2 - Participants |
|  | Log file analysis | N/A |
|  | Registration | N/A |
| Analysis | Handling of incomplete questionnaires | Section: Study 2 - Participants |
|  | Questionnaires submitted with an atypical timestamp | Section: Study 2 - Participants |
|  | Statistical correction | Section: Data Analysis |
